# Non syndromic supernumerary teeth: management of two clinical cases

**DOI:** 10.11604/pamj.2018.29.163.14427

**Published:** 2018-03-19

**Authors:** Akram Belmehdi, Soukayna Bahbah, Karima El Harti, Wafaa El Wady

**Affiliations:** 1 The Department of Oral Surgery, Dental Center of Treatment and Diagnosis (Ibn Sina Hospital), Rabat, Morocco; 2 The Oral Surgery Department, Faculty of Dentistry of Rabat, Mohammed V University, Morocco

**Keywords:** Supernumerary teeth, hyperdontia, impacted teeth, surgical management, radiographic images

## Abstract

Supernumerary teeth are extra teeth or tooth-like structures. Single, double, or multiple teeth that occur in one or both jaws may be erupted or unerupted and unilateral or bilateral. Supernumeraries are less common in primary dentition than in permanent dentition. The etiology of ST is still unknown. A number of theories have been postulated to try to explain their presence, including atavism (evolutionary throwback), tooth germ dichotomy, genetic and environmental factors, and hyperactivity of the dental lamina. However, all theories are hypothetical due to the inability to obtain sufficient embryologic material on their origin. The aim of this paper is two present two case reports of non syndromic supernumerary teeth in female patients and their management.

## Introduction

Supernumerary teeth (ST) or hyperdontia are uncommon alterations of development that may appear in either of the dental arches. While single tooth impaction is not uncommon, development of multiple impacted teeth is a rare condition and often found in association with syndromes or developmental anomalies such as cleidocranial dysplasia, Gardner's syndrome, trichorhino phalangic syndrome and cleft lip and palate [1]. Multiple ST in individuals with no other disease or syndrome is very rare [2]. In addition, they can occur unilaterally or bilaterally and can arise in the maxilla, mandible, or both [3]. Their overall prevalence ranges from 0.1% to 3.6%; however, it varies between the races [4]. Supernumerary teeth tend to adversely affect the neighboring dentition. They often hinder eruption and development of the permanent tooth related to them hence causing crowding, displacement, diastema, retention or delayed/ectopic eruption, root resorption, dental caries, periodontal lesions due to compression of adjacent roots and pulp necrosis and, in some cases, dentigerous cyst formation [5,6]. They cause delayed eruption or non-eruption of adjacent teeth and malformations of adjacent teeth at a rate of 21.6% [7]. The purpose of this paper is to present two cases of the patients who had supernumerary teeth without any associated syndrome or development anomaly.

## Patient and observation

**Figure 1:**
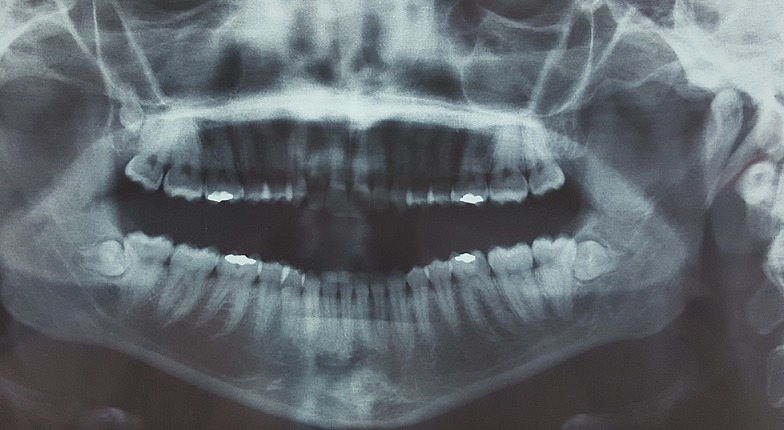
Orthopantomogram showing the presence of supernumerary teeth behind 18, 48 and 38

**Figure 2:**
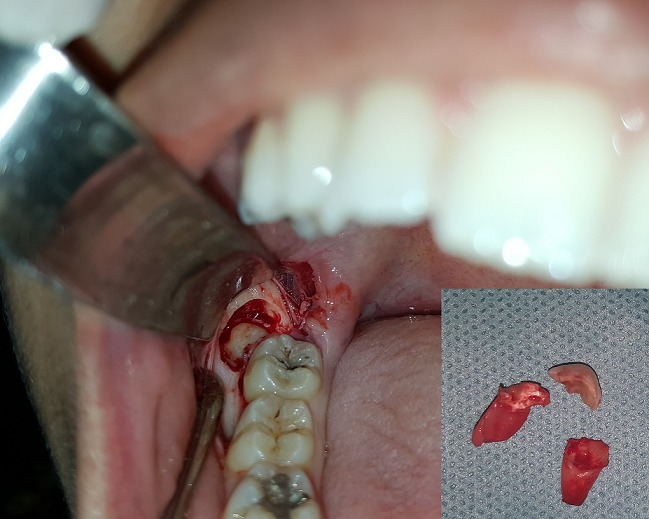
Peroperative view showing the surgical management of the right mandibular ST

**Case 1:** a 24-year-old female patient was referred to our department for the treatment of multiple impacted supernumerary teeth, which were detected on an orthopantomogram obtained at a dental clinic. An intraoral examination did not detect any abnormalities with regard to the size or shape of the patient's tooth crowns or the relationship between his dental age and chronological age, but a panoramic radiograph revealed three supernumerary teeth which were situated behind the 18, 48 and 38 (Figure 1). There was no any other specific oral finding and relevant familial history of dental abnormalities. The patient was educated about the presence of multiple supernumerary teeth and the extraction of the two mandibular supernumerary teeth was indicated before orthodontic treatment (Figure 2).

**Figure 3:**
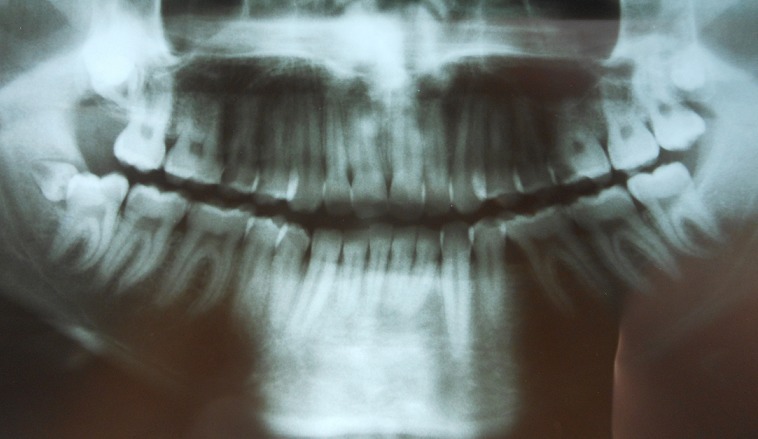
Orthopantomogram showing the presence of supernumerary teeth behind 18, 48 and 38

**Figure 4:**
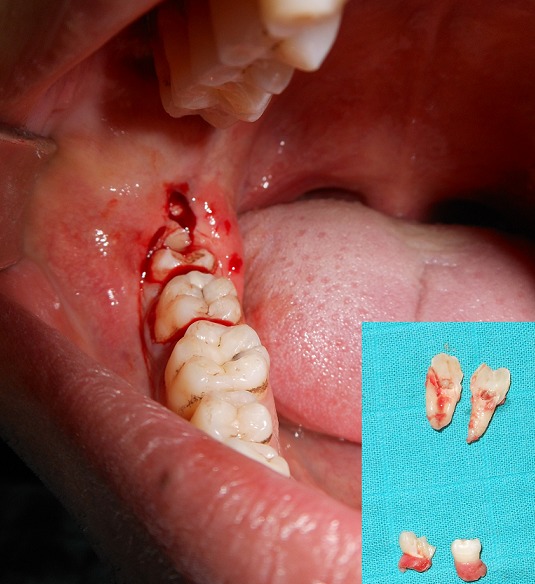
Peroperative view showing the surgical management of the right mandibular ST

**Case 2:** a 19-year-old girl was referred to our department because of a recurrent pericoronitis relevant to the lower right third molar 48. General physical and extra oral examination did not show any abnormality and medical/family history was non-contributory. Orthopantomogram has revealed a presence of 3 supernumerary teeth which were situated behind the 18, 28 and 48 (Figure 3). Surgical removal of the right mandibular supernumerary tooth was planned with extraction of the 48 (Figure 4) and the others ST will remain under surveillance following the patient decision.

## Discussion

Supernumerary tooth in normal dentition is not an unusual phenomenon but the presence of multiple supernumerary teeth in individuals without any syndromic disorder is not common. Literature shows that single supernumerary tooth is the most common [3,8] and they frequently locate in the anterior maxilla, specifically the midline. In contrast, multiple supernumerary teeth have been found to occur more frequently in the premolar region [4,8]. The literature shows that 76 to 86% of non-syndromic cases involve only one supernumerary tooth and that 12 to 23% of cases present two ST [2,3,9,10]. Analysis of ST cases shows that the variation is small, with the presence of one or two ST being more frequent. A single ST occurs in 72 to 77% of cases, two occur in 18 to 27% and three in only 1 to 5% [2,3,11]. The incidence of ST is generally higher in men, affecting premolars in approximately 10% of cases and almost 75% of these cases occur in the mandible [2,12]. Only 1% of non syndromic cases present multiple ST, which occur more frequently in the area of the mandibular premolars and in the anterior region [13-15]. The precise etiology of supernumerary teeth remains unclear. However, several theories have been postulated to explain their presence. These theories include atavism (evolutionary throwback) or phylogenetic theory, tooth germ dichotomy, hyperactivity of the dental lamina and genetic and environmental factors [16].

Orthopantomogram has been the modality of choice for investigating the status of supernumerary teeth, but the introduction of cone-beam computed tomography (CBCT) to radiographic technology has proved as the most effective three dimensional means of examining dental and associated osseous structures [7]. In our cases, we used orthopantomogram which has shown us the anatomic situations of ST. Regarding management issues of supernumerary teeth, different management options are available for patients with multiple hyperdontia not associated with complex syndromes. If the teeth are asymptomatic with no radiographic evidences of any pathologies and not likely to interfere with orthodontic tooth movement, (location beyond teeth apices) they can be monitored with periodic radiographic examination. But if the patient does not want to risk any complications, considerations can be given to extraction. If associated with roots of permanent teeth, waiting till the completion of root development should be considered to minimize the chances of root damage. If the supernumerary teeth are associated with any sort of complications like cysts or tumors, obstruction to normal teeth eruption, hindrance to orthodontic tooth movement and unaesthetic appearance, extraction is a logical management in those cases [8,17]. In our cases, we have decided to extract supernumerary to optimize orthodontic treatment (case 1) and following third molar complication (case 2) and we agreed with the patients to keep the other supernumerary tooth and put it under periodic monitoring.

## Conclusion

The key of a successful management of patients presented with supernumerary teeth requires a multidisciplinary approach and should form the part of a structured treatment plan in cooperation with pediatric dentistry, orthodontists and oral surgeons.

## Competing interests

The authors declare no competing interests.
